# Ochratoxin A induces liver inflammation: involvement of intestinal microbiota

**DOI:** 10.1186/s40168-019-0761-z

**Published:** 2019-11-28

**Authors:** Wence Wang, Shuangshuang Zhai, Yaoyao Xia, Hao Wang, Dong Ruan, Ting Zhou, Yongwen Zhu, Hongfu Zhang, Minhong Zhang, Hui Ye, Wenkai Ren, Lin Yang

**Affiliations:** 1 Guangdong Provincial Key Laboratory of Animal Nutrition and Regulation, College of Animal Science, South China Agricultural University, Guangzhou, 510642 China; 2 Institute of Animal Science, Guangdong Academy of Agricultural Sciences, Key Laboratory of Animal Nutrition and Feed Science (South China) of Ministry of Agriculture, Guangdong Key Laboratory of Animal Breeding and Nutrition, Guangzhou, 510640 China; 3 Guelph Food Research Center, Agriculture and Agri-Food Canada, Guelph, N1G 5C9 Canada; 4 State Key Laboratory of Animal Nutrition, Institute of Animal Sciences, Chinese Academy of Agricultural Sciences, Beijing, 100193 China

**Keywords:** Ochratoxin A, Intestinal microbiota, LPS, Liver inflammation, Fecal microbiota transplantation

## Abstract

**Background:**

Ochratoxin A (OTA) is a widespread mycotoxin and induces liver inflammation to human and various species of animals. The intestinal microbiota has critical importance in liver inflammation; however, it remains to know whether intestinal microbiota mediates the liver inflammation induced by OTA. Here, we treated ducklings with oral gavage of OTA (235 μg/kg body weight) for 2 weeks. Then, the microbiota in the cecum and liver were analyzed with 16S rRNA sequencing, and the inflammation in the liver was analyzed. To explore the role of intestinal microbiota in OTA-induced liver inflammation, intestinal microbiota was cleared with antibiotics and fecal microbiota transplantation was conducted.

**Results:**

Here, we find that OTA treatment in ducks altered the intestinal microbiota composition and structure [e.g., increasing the relative abundance of lipopolysaccharides (LPS)-producing *Bacteroides*], and induced the accumulation of LPS and inflammation in the liver. Intriguingly, in antibiotic-treated ducks, OTA failed to induce these alterations in the liver. Notably, with the fecal microbiota transplantation (FMT) program, in which ducks were colonized with intestinal microbiota from control or OTA-treated ducks, we elucidated the involvement of intestinal microbiota, especially *Bacteroides*, in liver inflammation induced by OTA.

**Conclusions:**

These results highlight the role of gut microbiota in OTA-induced liver inflammation and open a new window for novel preventative or therapeutic intervention for mycotoxicosis.

## Background

Mycotoxins are secondary metabolites produced by fungal genera (e.g., *Aspergillus*, *Penicillium*, and *Fusarium*) and are the most common natural food contaminants in human and animal diets, such as cereals and animal forages [1–3]. Ochratoxin A (OTA), the most prevalent and relevant fungal toxin produced by *Aspergillus* species and *Penicillium* species [4], is found to be one of the most common contaminants in cereals, coffee, wine, dried fruits and nuts, meat products [5], herbal medicines [6–8], food coloring agents [9], and even in bottled water [10]. OTA induces diverse toxic effects in host, including carcinogenic [11], hepatotoxic [12], nephrotoxic [13], and immunotoxic [14, 15]. OTA is metabolized and accumulated mainly in the liver and kidney; thus, the liver and kidney are the key target organs for OTA to exert its toxic effects [16, 17]. Previous studies have found that OTA induces inflammation and even cancer in the liver [12, 18–20]. Notably, OTA induces inflammation through the toll-like receptor (TLR)-4/myeloid differentiation factor (MyD) 88 signaling pathway [21].

Indeed, the absorption rate of OTA varies from animals to human (e.g., 66% in pigs, 56% in rabbits, and 40% in chicken) [22]. Intestinal barrier is the first line of host defense against encroaching commensal bacteria, invading enteric pathogens and natural toxins [23]. Numerous studies have shown that OTA disrupts intestinal barrier function, thereby inducing extraintestinal organ (e.g., liver) inflammation [24, 25]. Intestinal microbiota highly shapes the intestinal barrier function and the physiological function of extraintestinal organs [26]. Interestingly, the recent investigations showed that intestinal dysbiosis is tightly associated with the onset of hepatic inflammation and injury [27, 28]. Notably, OTA treatment alters intestinal microbiota in rats by changing the relative abundance of *Bacteroidaceae* and *Lactobacillaceae* [29]. However, whether OTA-induced liver inflammation involving in intestinal microbiota remains largely unknown.

Therefore, this study was conducted to explore the underlying mechanism of intestinal microbiota and bacterial translocation in the liver inflammation induced by OTA in ducks. The ducklings were used in this study since infants and young animals are more sensitive to OTA than matures due to their incomplete development of organs [30, 31], especially for duckling which serves as the most sensitive species by oral gavage OTA [32–35].

## Results

### Oral OTA gavage alters cecum microbiota composition and promotes cecum LPS biosynthesis in ducks

To explore the effects of oral OTA gavage on 21-day ducks, OTA residue, feed intake, final weight, weight gain, and feed/gain ratio were monitored during the experiment. The OTA residue was found in different organs, including the kidney, liver, muscle, and intestinal tissues (Additional file 1: Figure S1A). OTA had little effects on the growth performance (Additional file 1: Figure S1B–E), and showed little effects on the relative weight of organs, except the liver (Additional file 1: Figure S1F–H).

**Fig. 1:**
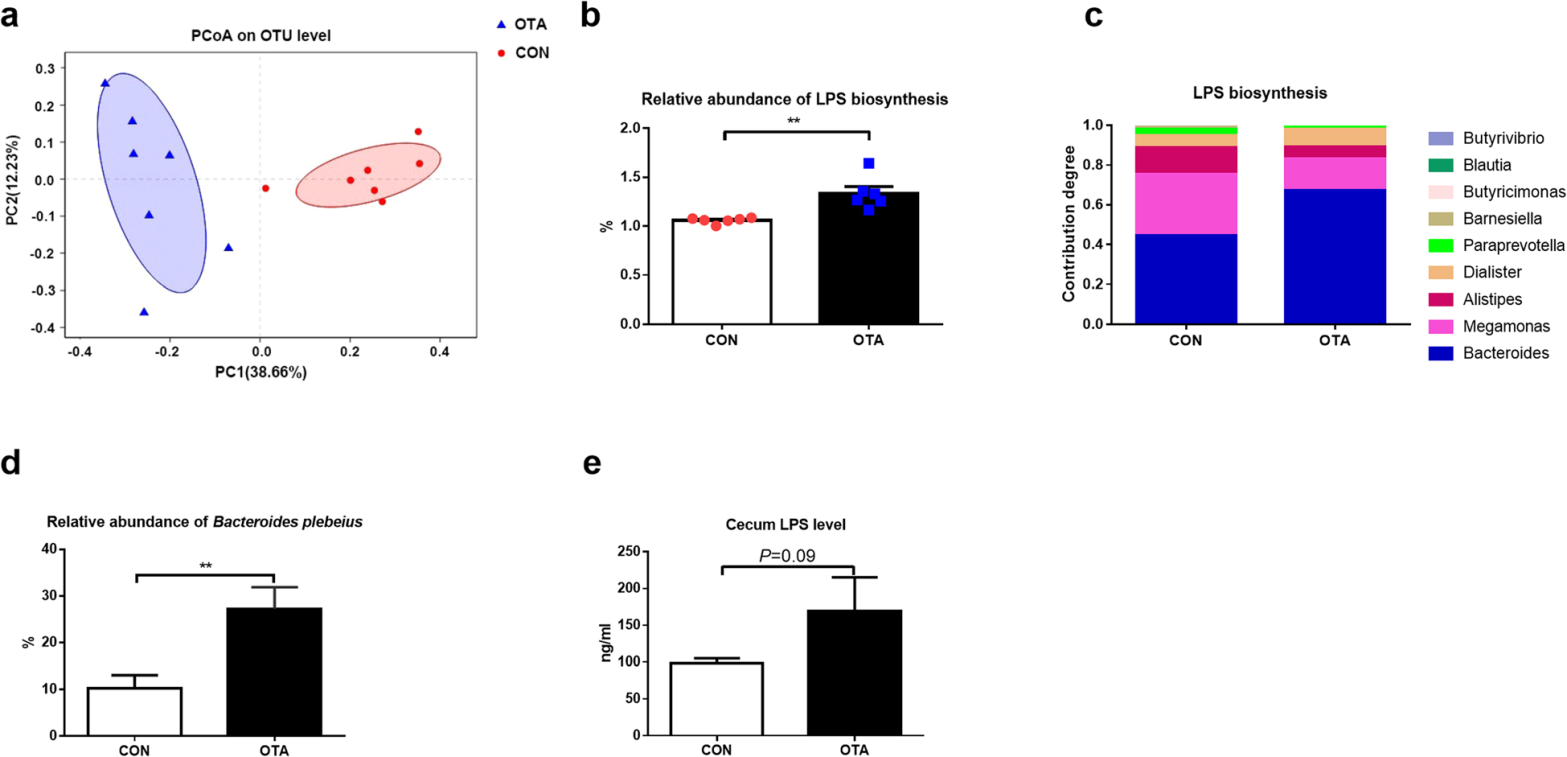
The composition and metagenomic function prediction of cecum microbiota after oral OTA treatment in ducks. **a** Principal component analysis of microbial communities in the cecum from CON and OTA. Plots are based on Bray-Curtis dissimilarities of 16S-based OTUs (*n* = 7). **b** Genes and gene families associated with LPS biosynthesis in different groups (*n* = 6, data shown as mean with SEM). **c** The contribution of different bacterial classes to LPS biosynthesis (*n* = 6). **d** Effect of OTA on the relative abundance of *Bacteroides plebeius *(*n* = 7). **e** The effect of OTA on cecum LPS level in ducks (*n* = 6, data shown as mean with SEM). For **b**, **d**, and **e**, data were analyzed with unpaired *t* test, **P < 0.01

To explore the effect of OTA on intestinal microbiota, cecum microbiota of ducks was analyzed by sequencing the cecum bacterial 16S rRNA V3 + V4 region and metagenomics. PCoA analysis showed a clear separation between the cecum microbiota of ducks in CON and OTA group (Fig. 1a), demonstrating a strong effect of OTA on cecum microbiota. OTA also significantly reduced the richness (ACE index) and diversity (Shannon index) of cecum microbiota (Additional file 2: Figure S2A). Besides the difference in diversity, OTA increased the relative abundance of *Bacteroidetes* in the phylum (Wilcoxon rank-sum test, *P* = 0.01; Additional file 2: Figure S2B) and *Bacteroides* in the genus (Wilcoxon rank-sum test, *P* < 0.05; Additional file 2: Figure S2C). Metagenomic results showed that OTA increased gene and gene family with LPS biosynthesis (*P* < 0.01, Fig. 1b). With the analysis of contribution capacity of different strains to LPS biosynthesis by metagenomic sequencing, *Bacteroides* displayed the greatest contribution to LPS biosynthesis (Fig. 1c). Notably, the relative abundance of *Bacteroides plebeius* was higher in OTA group than those in CON group (Fig. 1d, Additional file 2: Figure S2D). To test the LPS biosynthesis ability, the LPS levels in the cecum were determined. The LPS level in OTA-treated ducks showed 1.5-fold higher than those ducks without OTA treatment (Fig. 1e). Collectively, OTA induces dysbiosis of the intestinal microbiota, especially increasing LPS-producing *Bacteroides.*

### OTA alters microbiota in the liver of ducks

**Fig. 2:**
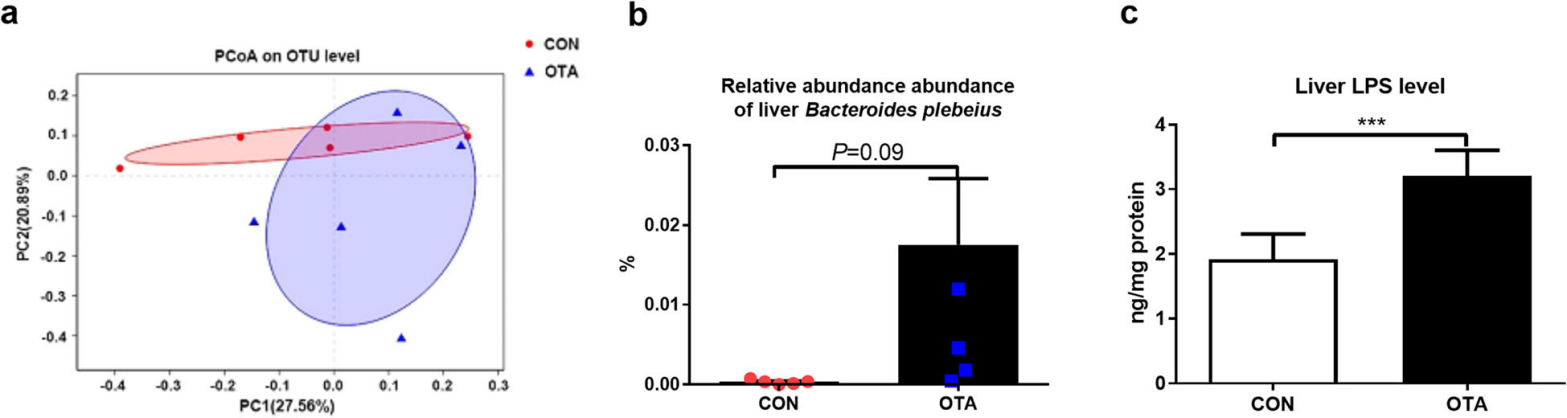
The composition of liver microbiota after oral OTA treatment in ducks. **a** Principal component analysis of microbial communities in the liver from CON and OTA (*n* = 5). **b** Effect of OTA on the relative abundance of *Bacteroides plebeius*. **c** Effect of OTA on liver LPS level (*n* = 5, mean with SEM). Data in **b** and **c** were analyzed with unpaired *t* test, ***P < 0.001

Besides its effect on cecum microbiota, OTA also lowered the mRNA expression and protein abundance of tight junction proteins (TJP1 and Occludin) (Additional file 3: Figure S3), suggesting that OTA enhances the cecum permeability, which is a widely accepted conclusion from previous studies [24, 25]. Based on these results, we hypothesized that LPS-producing *Bacteroides* may enter the liver through the leaky gut after OTA treatment. To explore this possibility, the microbiota in the liver was analyzed. PCoA analysis showed the difference about the liver microbiota in the CON and OTA groups (Fig. 2a). Although OTA had no effect on richness and diversity of liver microbiota in ducks (Additional file 4: Figure S4A), OTA significantly increased the relative abundance of *Bacteroidetes* in the phylum level (Student’s *t* test, *P*=0.02), the relative abundance of *Bacteroides* (Student’s *t* test, *P* < 0.01) at the genus level, and the relative abundance of *Bacteroides plebeius* at the species level (Fig. 2b and Additional file 4: Figure S4B–D). Interestingly, we also found that OTA treatment significantly increased the level of LPS in the liver (Fig. 2c). Collectively, OTA increases the relative abundance of LPS-producing *Bacteroides* and the level of LPS in the liver of ducks.

### Oral OTA promotes liver inflammation

**Fig. 3:**
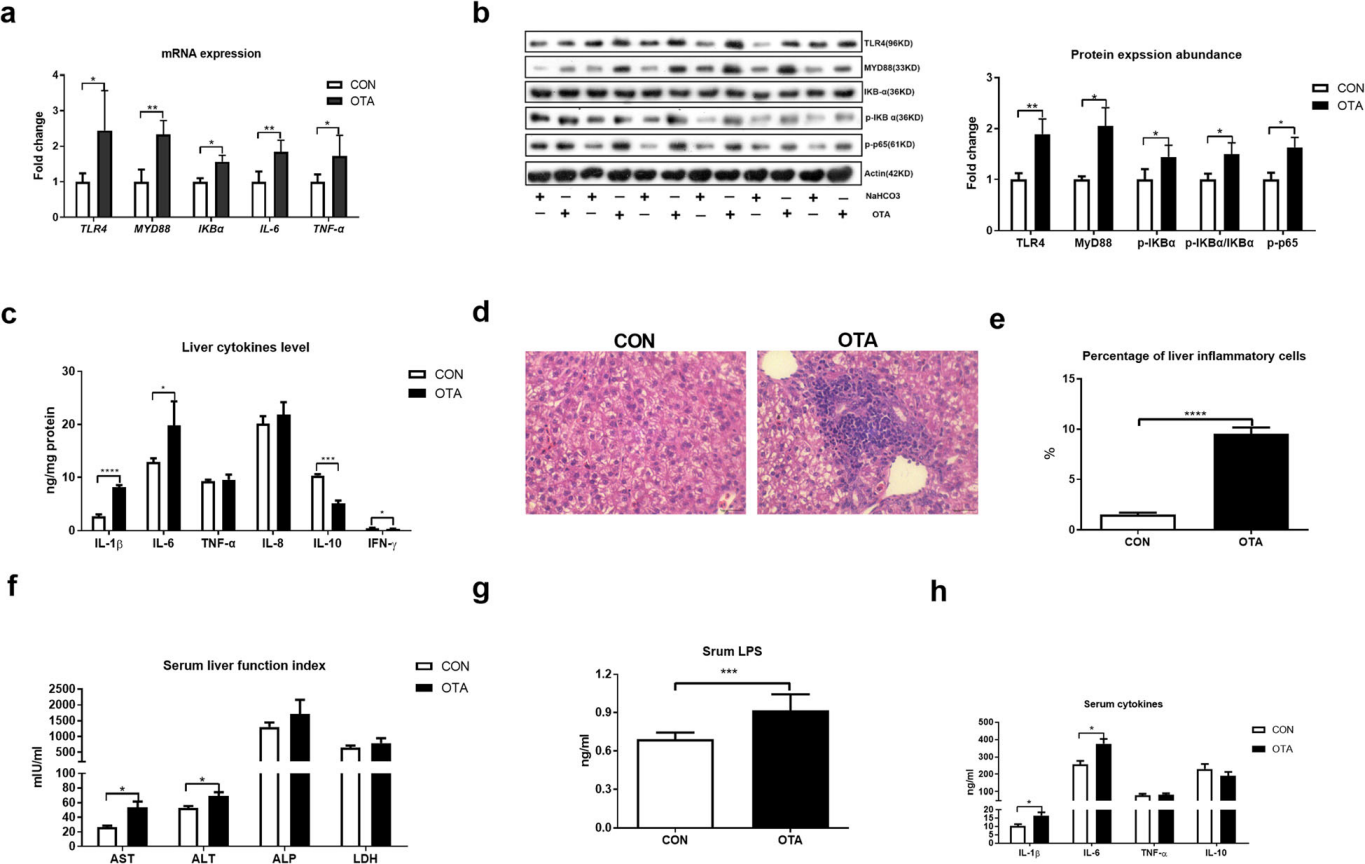
OTA promotes liver inflammation. **a** Relative mRNA expressions of TLR4, MYD88, IKBα, IL-6, and TNF-α in the liver after OTA oral gavage (*n* = 6, mean with SEM). **b** Relative protein abundances of TLR4, MYD88, p-IKBα, p-IKBα/IKBα, and p-p65 in the liver after OTA oral gavage (*n* = 6, mean with SEM). **c** Effect of OTA on levels of liver cytokines, including IL-1β, IL-6, TNF-α, IL-8, IL-10, and IFN-γ (*n* = 6, mean with SEM). **d** Representative images of H&E-stained liver sections in CON and OTA group (magnification × 400, scale bar 100 μm, *n* = 6). **e** Statistical analysis of the percentage of inflammatory cells in different groups shown in **d** (*n* = 6, mean with SEM). **f** Effect of OTA on serum levels of AST, ALT, ALP, and LDH (*n* = 6, mean with SEM). **g** Serum LPS level with or without OTA treatment (*n* = 6, mean with SEM). **h** Effect of OTA on serum levels of IL-1β, IL-6, TNF-α, and IL-10 (*n* = 6, mean with SEM). Data in **a**, **b**, **c**, **e**, **f**, **g**, and **h** were analyzed with unpaired *t* test, *P < 0.05; **P < 0.01, *** *P* < 0.001, *P*<0.0001

Based on the higher relative weight of the liver and level of LPS in the liver after OTA treatment, we then analyzed the inflammatory responses in the liver. The activation of the TLR-4 signaling pathway was activated after OTA treatment based on the higher mRNA expression and protein abundance of TLR4 Myd88 and p-p65 and higher ratio of p-IKBα/IKBα (Fig. 3a, b). OTA treatment also promoted the mRNA expression and secretion of inflammatory cytokines, including IL-1β and IL-6 (Fig. 3a, c). Notably, the level of anti-inflammation cytokine IL-10 was lowered after OTA treatment (Fig. 3c). Histological analysis also indicated that OTA induced inflammation in the liver based on extensive inflammatory cell infiltration in OTA-treated liver (Fig. 3d, e). The inflammation in the liver was also supported by the evidence that OTA treatment increased the activities of serum AST and ALT and the levels of serum LPS, IL-1β, and IL-6 (Fig. 3f, h). Together, OTA promotes liver inflammation in ducks.

### OTA has little effect on cecum microbiome in antibiotics-treated ducks

**Fig. 4:**
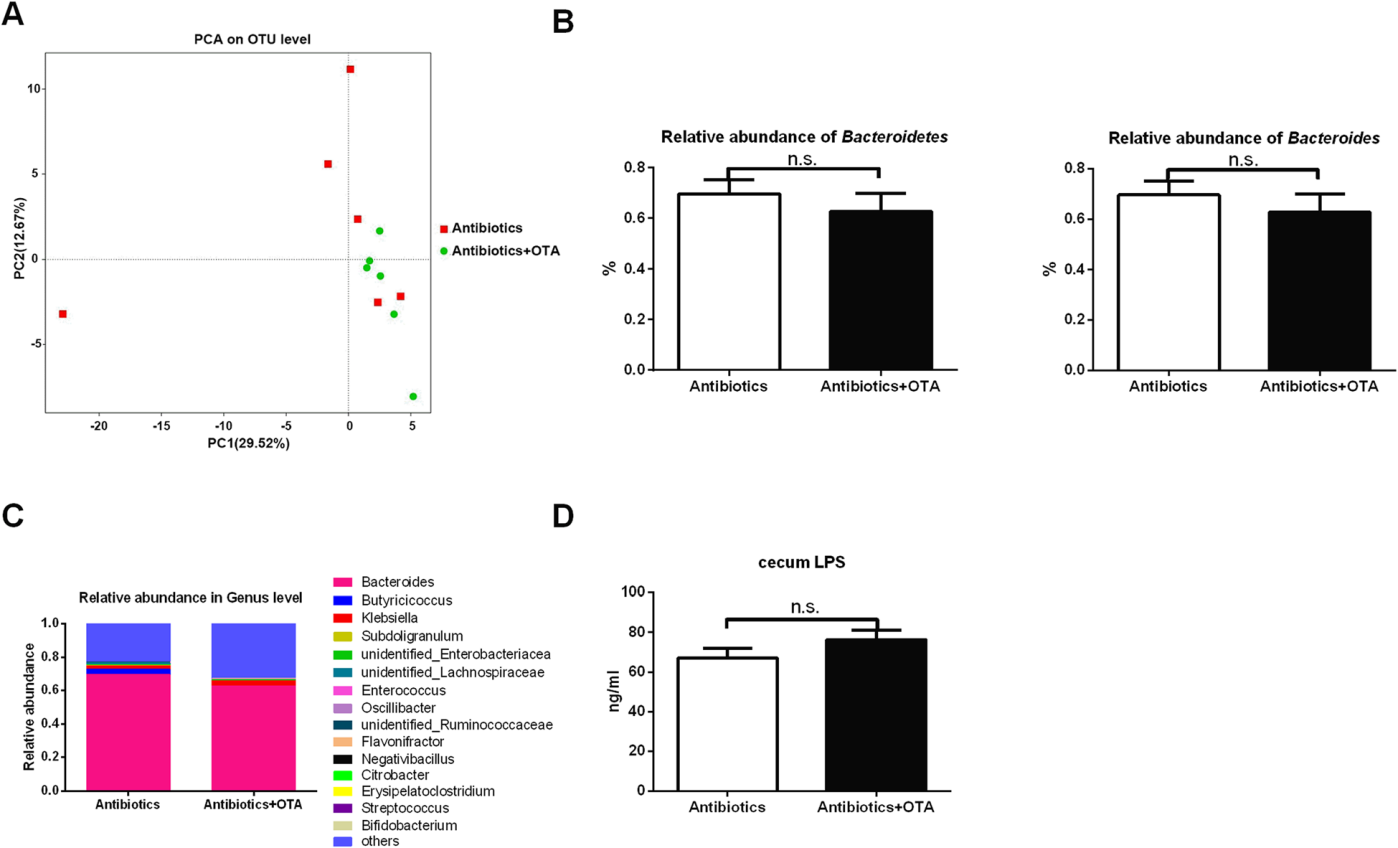
OTA has little effect on microbiome composition and LPS level in the cecum after antibiotics treatment. **a** PCoA of microbiota communities in the cecum between two groups (*n* = 6). **b** The relative abundance of *Bacteroidetes* (at the phylum level) and *Bacteroides* (at the genus level) (*n* = 6, mean with SEM). **c** Relative abundance of top 15 genus in each group (*n* = 6). **d** Effect of OTA on cecum LPS level in antibiotics-treated ducks (*n* = 6, mean with SEM). Data in **b** and **d** were analyzed with unpaired *t* test; n.s., not significant

We have shown that OTA induces dysbiosis of intestinal microbiota and liver inflammation in ducks; thus, we hypothesized that OTA promotes the liver inflammation through intestinal microbiota. To clear the intestinal microbiota, ducks were treated with antibiotic mixtures, including streptomycin, ampicillin, and neomycin. Antibiotic treatment showed little effect on OTA residue in ducks, and OTA had little effects on growth performance, organ indexes, and relative weight and length of the intestine in antibiotics-treated ducks (Additional file 5: Figure S5A–H). Notably, OTA had little effects on the relative weight of the liver in antibiotics-treated ducks (Additional file 5: Figure S5F). PCoA analysis showed the similarity of cecum microbiota between two groups (Fig. 4a), and the diversity of cecum microbiota between two groups were similar (Additional file 6: Figure S6A), suggesting OTA has little effect on intestinal microbiota in antibiotics-treated ducks.

Antibiotic treatment extremely decreased the absolute abundance of *Bacteroidetes* (Additional file 6: Figure S6B, *P* < 0.0001) and *Bacteroides* (Additional file 6: Figure S6B, *P* < 0.001) compared with ducks without antibiotic treatment, while OTA showed little effect on the relative and absolute abundance of *Bacteroidetes* and *Bacteroides* in antibiotics-treated ducks (Fig. 4b and Additional file 6: Figure S6B). Moreover, OTA had little effect on the composition of cecum bacteria at the phylum, genus, and species level (Fig. 4c and Additional file 6: Figure S6C–D). Likewise, OTA had little effect on the cecum LPS level (Fig. 4d). These results indicated that OTA has little effect on cecum microbiota in antibiotics-treated ducks.

### OTA failed to promote liver inflammation in antibiotics-treated ducks

**Fig. 5:**
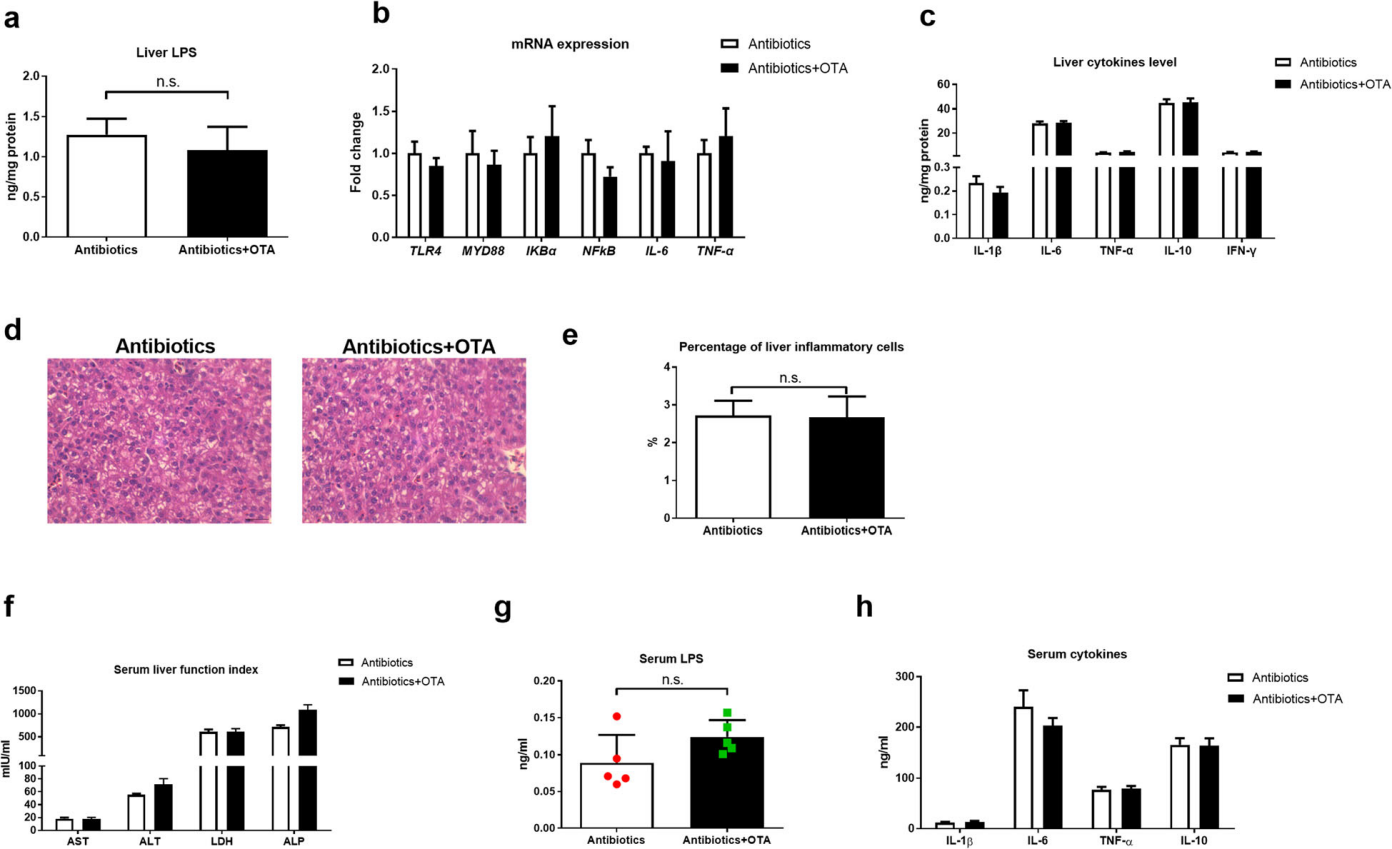
OTA fails to promote liver inflammation in antibiotics-treated ducks. **a** Liver LPS level in different groups (*n* = 6, mean with SEM). **b** Relative mRNA expressions of TLR4, MYD88, IKBα, IL-6, and TNF-α in the liver of antibiotics-treated ducks with or without OTA (*n* = 6, mean with SEM). **c** Levels of IL-1β, IL-6, TNF-α, IL-8, IL-10, and IFN-γ in the liver of antibiotics-treated ducks with or without OTA (*n* = 6, mean with SEM). **d** Representative images of H&E-stained liver sections (magnification × 400; scale bar 100 μm; *n* = 6). **e** Statistical analysis of the percentage of inflammatory cells in different groups shown in **d** (*n* = 6, mean with SEM). **f** Effect of OTA on serum levels of AST, ALT, ALP and LDH in antibiotics-treated ducks (*n* = 6, mean with SEM). **g** Serum LPS level with or without OTA treatment in antibiotics-treated ducks (*n* = 6, mean with SEM). **h** Effect of OTA on serum levels of IL-1β, IL-6, TNF-α, and IL-10 (*n* = 6, mean with SEM). Data were analyzed with unpaired *t* test. n.s., not significant

Besides the results that OTA had little effects on cecum microbiome in antibiotics-treated ducklings, OTA had little effects on the mRNA expression and protein abundance of TJP1 and Occludin in antibiotics-treated ducks (Additional file 7: Figure S7). Notably, OTA had little effects on the levels of LPS in liver and the liver inflammation in antibiotics-treated ducks, including mRNA expression of TLR4 and Myd88, secretion of inflammatory cytokines, and inflammatory cell infiltration (Fig. 5a–e). OTA also had little effects on the activities of serum AST and ALT and levels of serum LPS and inflammatory cytokines (Fig. 5f–h). Together, OTA fails to promote liver inflammation in antibiotics-treated ducks.

### OTA-originated microbiota promotes the accumulation of LPS-producing *Bacteroides* and LPS in the cecum

**Fig. 6:**
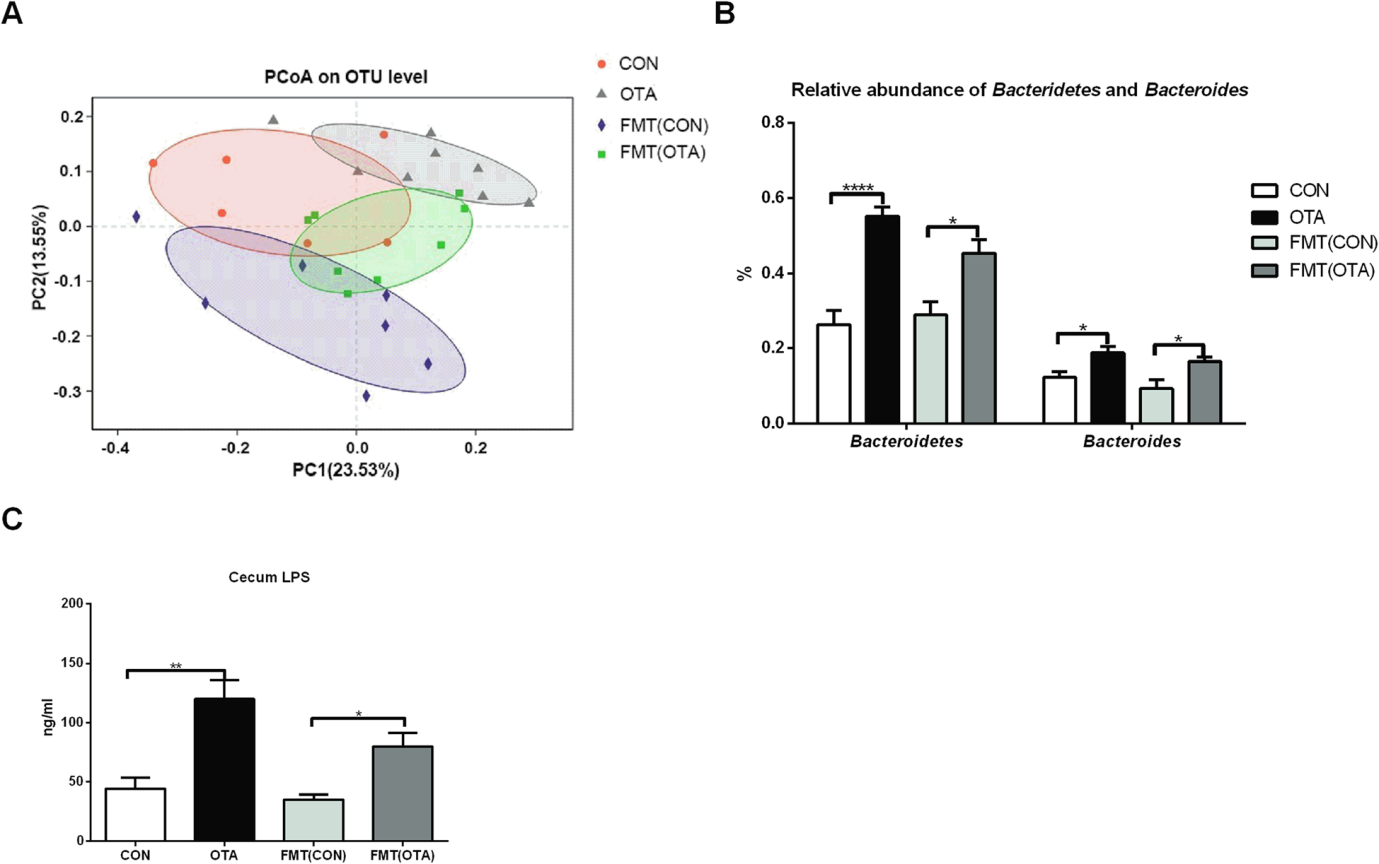
Gut microbiota composition and LPS level after intestinal microbiota transplantation. **a** Principal-coordinate analysis (PCoA) of cecum microbiota for transplant donors and recipients. Each circle represents a group. **b** Relative abundance of *Bactroidetes* and *Bacteroides* for transplant donors and recipients (*n* = 7, Mean with SEM). **c** Cecum LPS levels for different groups (*n* = 5, mean with SEM). Data in **b** and **c** were analyzed by one-way ANOVA with Bonferroni’s multiple comparisons test, *P < 0.05, **P < 0.01, ****P < 0.0001. FMT (CON): ducks received the CON group fecal microbiota. FMT (OTA): ducks received the OTA group fecal microbiota

To further verify the role of gut microbiota in OTA-induced liver inflammation, we transplanted fecal microbiota from oral OTA-treated ducks into antibiotics-treated ducks. As a control, the fecal microbiota from normal ducks was also transplanted into antibiotics-treated ducks. After OTA-originated microbiota transplantation, the load of OTA in the cecum was much lower than those with OTA treatment, and it was hardly detected in the liver (Additional file 1: Figure S1A, Additional file 5: Figure S5A and Additional file 8: Figure S8A). Although OTA-originated microbiota had little effect on the bodyweight of recipient ducks, it significantly enhanced the relative weight of the liver (Additional file 8: Figure S8B–C). Considering the OTA residue in OTA-originated fecal microbiota, we analyzed the OTA residue in OTA-originated microbiota. Recipient duck after OTA-originated microbiota transplantation received OTA at a dosage of 2 ng/kg body weight. Notably, OTA failed to induce liver inflammation in ducks even with a dosage of 60 μg/kg body weight (Additional file 8: Figure S8D), ruling out the possibility that the liver inflammation in recipient ducks is from OTA in OTA-originated fecal microbiota. Interestingly, OTA-originated microbiota significantly altered the microbiota in the cecum and enhanced the relative abundance of *Bactroidetes* and *Bacteroides*, as well as the level of LPS (Fig. 6a–c, Additional file 9: Figure S9). These results suggest that OTA-originated microbiota promotes the accumulation of LPS-producing *Bacteroides* and LPS in the cecum.

### OTA-originated microbiota induces liver inflammation

**Fig. 7:**
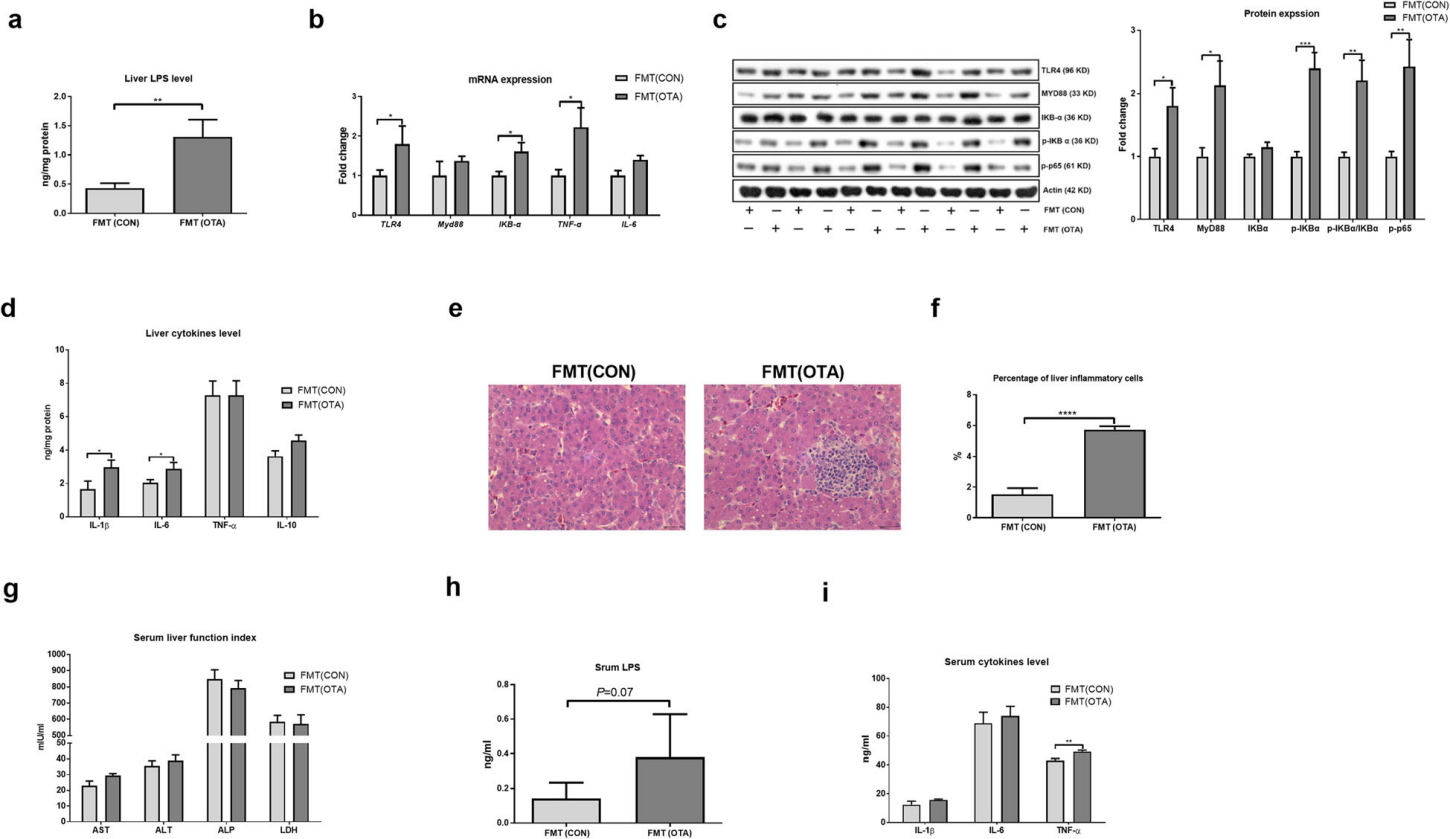
OTA-originated microbiota induces liver inflammation. **a** Liver LPS level in FMT (CON) and FMT (OTA) ducks. **b** Relative mRNA expressions of TLR4, MYD88, IKBα, IL-6, and TNF-α in the liver after FMT (*n* = 6, mean with SEM). **c** Relative protein abundance of TLR4, MYD88, p-IKBα, p-IKBα/IKBα, and p-p65 in the liver after FMT (*n* = 6, mean with SEM). **d** Effect of FMT on the liver levels of IL-1β, IL-6, TNF-α, and IL-10 (*n* = 6, mean with SEM). **e** Representative H&E-stained liver sections. **f** Statistical analysis of the percentage of inflammatory cells in different groups shown in **e** (*n* = 6, mean with SEM). **g** Serum levels of AST, ALT, ALP, and LDH in different groups (*n* = 6, mean with SEM). **h** Serum LPS level in different FMT groups (*n* = 6, mean with SEM). **i** Serum levels of IL-1β, IL-6, and TNF-α after FMT (*n* = 6, mean with SEM). FMT (CON): ducks received the CON group fecal microbiota. FMT (OTA): ducks received the OTA group fecal microbiota. Data were analyzed with unpaired *t* test, *n* = 6. *P < 0.05; **P < 0.01, *** *P* < 0.001, ****P<0.0001

To explore the role of gut microbiota in OTA-induced liver inflammation, the expression of tight junctions in the cecum and liver inflammation was analyzed. OTA-originated microbiota lowered the mRNA expression and protein abundance of TJP1 and Occludin in recipient ducks (Additional file 10: Figure S10). Interestingly, OTA-originated microbiota promoted the liver level of LPS and liver inflammation in recipient ducks, including mRNA expression of TLR4 and TNF-α, protein abundance of TLR4, Myd88 and p-p65, ratio of p-IKBα/IKBα, secretion of IL-1β and IL-6, and inflammatory cell infiltration (Fig. 7a–f). OTA-originated microbiota also enhanced the levels of LPS and TNF-α in the serum (Fig. 7g–i). Together, OTA-originated microbiota induces liver inflammation in recipient ducks.

## Discussion

OTA is a major food-contaminating mycotoxin and shows nephrotoxic, hepatotoxic, teratogenic, immunotoxic, and carcinogenic effects within phylogenetically distant organisms from animals to human [36]. Intestinal microbiota represents a crucial bridge between environmental substances and host health. Indeed, in addition to the important roles in maintaining gastrointestinal tract homeostasis, intestinal microbiota also affects multitudinous physiological functions of other visceral organs (e.g., brain, liver, and lung) and is involved in mediating the pathogenesis of different diseases in these organs [37–42]. Although previous investigations have demonstrated that OTA reduces the relative abundance of beneficial microbes (e.g., *Lactobacillus* and *Bifidobacteria*) [43], shapes the diversity of the intestinal microbiota, and increases the fecal total facultative anaerobes in rats [44], whether OTA-induced injuries in extraintestinal organs (e.g., liver and kidney) through intestinal microbiota remains to be revealed.

We found that OTA alters the intestinal microbiota composition in ducks, including the decrease in the richness and diversity of microbiota, and the relative abundance of *Firmicutes*, as well as the increase of the relative abundance of *Bacteroidetes* and *Bacteroides*. The relative abundance of *Bacteroides* accounts for 32.69% of the bacterial abundance in the normal ducks, but up to 52.99% after OTA treatment. Guo et al [44] also found that OTA decreases the within-subject diversity of the intestinal microbiota and induces changes in functional genes of gut microbiota, like signal transduction, carbohydrate transport, and amino acid transport system in OTA-treated rats. These results indicate that OTA changes intestinal microbiota composition in various animal species. Given intestinal microbiota metabolism has various physiological functions in the intestine and extraintestinal organs, thus, it is interesting to investigate whether OTA affects the intestinal metabolites (e.g., acetate, propionate, and butyrate) in the future.

Although the *Bacteroides* (Gram-negative bacteria) is often associated with the leanness and other desirable health traits [45, 46], some of its strains (e.g., *Bacteroides fragilis, Bacteroides vulgatus*, and *Bacteroides dorei*) have been linked to abdominal infections, metabolic disease, and inflammation [47, 48]. LPS or endotoxin is the main component of Gram-negative bacteria and induces inflammatory responses after across the mucosa [49–51]. After translocation in the portal circulation through enterohepatic recycling, LPS or the translocated microbiota, such as *L. monocytogenes*, contributes to inflammation through the engagement of various pattern-recognizing receptors (PRRs), including TLR4 or activation of NF-κB [52, 53]. Mechanistically, TLR4 (special receptor for LPS) leads to a cascade of the phosphorylation of mitogen-activated protein kinases (MAPKs), activation of NF-κB signaling, and expressions of various inflammatory cytokines, chemokines, and adhesion molecules [52].

A leaky gut permits the passage of microbial products, such as LPS, across the mucosa, which manifests as a moderate increase in the plasma LPS concentration [54, 55]. Indeed, when the gut barrier is compromised, microorganisms and microorganism-derived molecules can translocate to the liver, causing inflammation and hepatic injury [56]. Previous studies also have demonstrated that OTA drives intestinal barrier dysfunction and induces inflammation [57, 58]. In this study, we found that OTA treatment triggers intestinal barrier dysfunction and increases subsequent systemic LPS levels and inflammatory responses in the liver of ducks. Besides, studies have shown that anaerobic bacteria do not readily translocate, whereas aerobic Gram-negative bacteria translocate easily and even across an intact intestinal epithelium barrier [59, 60]. We detected *Bacteroides*, which were dominated in intestinal microbiota, still occupied the main status in the liver microbiome of the OTA group, indicating that they were probably from the intestinal microbiota by a leaky gut. Given the findings that OTA increases the relative abundance of gene and gene family associating with LPS biosynthesis, and that there is a positive correlation between LPS biosynthesis and *Bacteroides*; thus, OTA promotes liver inflammation may through intestinal LPS-producing *Bacteroides.* It is worth mentioning that OTA increases the relative weight of the liver [57, 61, 62]. Interestingly, in the current study, we also find that OTA treatment increases the liver weight of ducks. The possible explanations include (1) as the main target organ for OTA, OTA causes hepatocyte excessive proliferation, swelling, and hyperemia [63]; (2) OTA induces compensatory increment of connective tissue of liver [64]; and (3) OTA induces the infiltration of immune cells during the inflammation. It is interesting to investigate some opening questions, like the underlying mechanisms by which OTA promotes the relative percentage of LPS-producing *Bacteroides*.

Notably, OTA shows little effect on the accumulation of LPS in the cecum, serum, and liver and the activation of the TLR4 signaling pathway, as well as the liver inflammation in antibiotics-treated ducks. Additionally, in order to explore the contribution of the intestinal microbiome to OTA-induced liver inflammation, we carried out the fecal microbiota transplantation (FMT) experiment. Microbial transplantation provides an excellent way to demonstrate the role of gut microbiota in host defense mechanisms under a similar genetic background [65]. The previous study has also shown that FMT has a very high success rate in altering metabolic phenotypes, curing diseases, and affecting the host immune status [66–69]. In our study, antibiotics-treated ducks colonized with the fecal microbiota from OTA-treated ducks show similar phenotypes (cecum microbiota composition, cecum LPS, intestinal permeability, and liver inflammation) with OTA-treated ducks. Altogether, based on these results, we conclude that the gut microbiota, especially LPS-producing *Bacteroides*, contributes to the liver inflammation. Indeed, the liver is the target of xenobiotics and performs xenobiotic metabolism [70]; thus, it is meaningful to investigate whether OTA affects liver biosynthetic (e.g., total protein and prothrombin) and metabolic (e.g., lipid metabolism) capacity directly and/or indirectly through intestinal microbiota. Likewise, it is also interesting to study whether the pathogenesis of OTA-induced disease in other organs (e.g., brain and kidney injury) depends on intestinal microbiota.

## Conclusions

Collectively, our study has identified a new mechanism linking the intestinal microbiota to OTA-induced liver inflammation. OTA alters cecum microbiota diversity and composition, leading to an increase in Gram-negative bacterial-derived LPS and entrance into the blood and liver through defective intestinal barrier, and ultimately facilitates the development of liver inflammation in ducks.

## Methods

### Animal and diet

All animal experiments were conducted according to the guidelines of Guangdong Province on the Review of Welfare and Ethics of Laboratory Animals, and approved by the Guangdong Province Administration Office of Laboratory Animals (GPAOLA). Male one-day-old Peking ducklings were purchased from Guiliu Poultry Co., Ltd. (Foshan, China). Animals were housed in stainless steel cages (five ducklings/cage) with free access to water and food, and kept under controlled room (temperature, maintained at 33 ± 1 °C for the first 3 days and then reduced by 2.5 ± 0.5 °C per week to a final temperature of 26 °C; relative humidity, 45%–60%; lighting, 24 h lighting with 10 Lux). Ducklings were fed a corn-soybean meal basal diet formulated to meet the nutritional requirements for starter ducks (National Research Council, 1994).

### OTA oral gavage for ducklings

One-day-old male Peking ducklings were divided randomly into CON and OTA group with 15 replicates per group. On day 8, ducklings in the OTA group were challenged once a day for 2 weeks by i.g gavage OTA (Pribolab, Qingdao, China) in doses of 235 μg/kg body weight with 0.1 M sodium bicarbonate. This dosage was selected according to the previous study, which could increase the relative weight of the liver, result in a clear sign of enterotoxity, and increase levels of the pro-inflammatory cytokines in duck jejunal mucosa [57]. After 2 weeks of OTA oral gavage, ducks were executed by bloodletting of the jugular vein (at 09:00). Individual blood samples were collected from the jugular vein, and serum samples were separated by centrifugation of blood at 1200×*g* for 10 min at 4 °C and stored at − 30 °C for analysis. For conventional analysis, the entire liver, duodenum, jejunum, ileum, cecum, and rectum were collected and weighed. The middle part of liver samples (about 1–2 cm) was collected after the phosphate-buffered saline (PBS; pH = 7.2–7.4) washing. The liver was fixed in fresh 4% paraformaldehyde for paraffin embedding. For molecular biological analysis, parts of the liver and cecal mucosa were snap-frozen in liquid nitrogen for mRNA and protein extraction for qRT-PCR and Western blotting. Cecum digesta were collected and stored at − 80 °C until further analyses. The body weights of the animals were regularly monitored during the treatment period.

### OTA oral gavage for antibiotics-treated ducklings

One-day-old Peking male ducklings were divided randomly into antibiotics and antibiotics + OTA groups with 15 replicates per group. All ducklings received a basic diet and drinking water containing streptomycin (1 g/L, Sigma), ampicillin (1 g/L, Sigma), and neomycin (1 g/L, Sigma) to clear intestinal bacteria, and drinking water was prepared daily [71]. The ducklings in the antibiotics + OTA group were treated similarly to the OTA group. After 2 weeks of OTA oral gavage, ducks were executed by bloodletting of the jugular vein (at 09:00) to collect the serum, liver, cecum mucosa, and cecum digesta for further analyses. The body weights of the animals were regularly monitored during the treatment period.

### Fecal microbiota transplantation

One-day-old Peking male ducklings were divided randomly into CON, OTA, FMT (CON), and FMT(OTA) groups with 15 ducklings per group (*n* = 15). FMT(CON) and FMT(OTA) groups received a basal diet and drinking water containing streptomycin (1 g/L), ampicillin (1 g/L), and neomycin (0.5 g/L) for 2 weeks to remove indigenous gut microorganisms. After this treatment, the antibiotics-containing water was replaced with the regular water, and the microbiota-depleted ducklings were transplanted with donor microbiota. For fecal microbiota transplantation, 10 g fresh fecal samples were collected from the CON and OTA groups and resuspended in 50 ml sterile anaerobic saline, vortexed for 3 min and allowed to settle by gravity for 2 min. Transplant into recipient ducklings was achieved by gavage with 10 ml/kg body weight of the supernatant from the fecal sample once a day for 2 weeks. Then, ducks per group were executed by bloodletting of the jugular vein (at 09:00) to collect the serum, liver, cecum mucosa, and cecum digesta for further analysis. The body weights of the animals were regularly monitored during the treatment period.

### Histopathology

After embedding in paraffin blocks, formalin-fixed liver tissues were sectioned to 6 μm thicknesses on a microtome (LeicaRM2235, Leica, Nussloch, Germany). The sections were placed on silicon-coated glass slides (Leica Biosystem, Richmond, IL, USA), dried, deparaffinized with xylene, and rehydrated in decreasing ethanol series. The sections were stained with 2.5% hematoxylin (Merck, Darmstadt, Germany) followed by counterstaining with 0.5% eosin (Sigma Aldrich, USA). Stained tissue sections were examined under an Olympus BX61 light microscope (Olympus, Tokyo, Japan) with × 200 magnification. Images were captured (scale bar=100 µm) using an Olympus DP70 digital camera (Olympus, Japan).

### Biochemical analysis

The organs were pretreated with 70% methanol, and the residual OTA was measured by an ELISA Kit (RNM98008, REAGEN, USA). The activity of serum aspartate aminotransferase (AST), alanine aminotransferase (ALT), alkaline phosphatase (ALP), and lactate dehydrogenase (LDH) and the levels of cytokines, including IL-1β, TNF-α, IL-6, and IL-10 in the liver and serum, were measured with ELISA Kit (Nanjing Jiancheng Bioengineering Institute, Nanjing, China) according to the manufacturer’s instructions. LPS levels were measured in the cecum, liver, and serum using ELISA Kit (Cloud-Clone Crop., Houston, USA).

### Western blotting for protein expression

Whole protein, including Occludin and TJP-1, which are responsible for maintaining the intestinal barrier function [72], and TLR4 and NF-κB signaling pathway, which are highly crucial for modulating inflammatory responses [52], from the liver and cecum mucous were lysed by RIPA lysis buffer (LifeTechnologies Inc., USA) supplemented with protease inhibitor cocktail (Roche, USA). The protein concentration in the tissue lysate was measured with BCA. Proteins were loaded onto the SDS-PAGE gel (BioRad) and electrophoresed and analyzed by WB using antibodies against TLR4 (BA1717, Boster, Wuhan, China), MYD88 (abs135682, Absin, Shanghai, China), IKBα (D120138, Sangon Biotech, Shanghai, China), p-IKBα (D151548, Sangon Biotech, Shanghai, China), p-p65 (HZ4902812, TW reagent, Shanghai, China), TJP-1 (mAb13663, Cell Signaling, USA), Occludin (ab167161, Abcam, USA), and actin (60008, Proteintech, USA). The bands were detected using the chemiluminescence kits (Amersham Biosciences, UK). Chemiluminescence was recorded with an Image Station 440CF, and results were analyzed with the 1DImage Software (Kodak Digital Science, Rochester, NY, USA).

### 16S rRNA amplicon sequencing, data processing, and analysis

DNA was extracted from the feces and liver using the E.Z.N.A.® soil DNA Kit (Omega Bio-tek, Norcross, GA, USA) according to the protocol for isolation of DNA. Illumina MiSeq sequencing and general data analyses were performed by a commercial company (Majorbio Bio-Pharm Technology, Shanghai, China). Because of initially low bacterial DNA concentrations in some samples, a nested PCR was applied to increase specificity and amplicon yield [73, 74]. The V3–V4 hypervariable regions of the bacteria 16S rRNA gene were amplified with primers 338F (5′-ACT CCT ACG GGA GGC AGC AG-3′) and 806R (5′-GGA CTA CHV GGG TWT CTA AT-3′) by thermocycler PCR system (GeneAmp 9700, ABI, USA). The PCR reactions were conducted using the following program: 3 min of denaturation at 95 °C, 27 cycles of 30 s at 95 °C, 30s for annealing at 55 °C, 45 s for elongation at 72 °C, and a final extension at 72 °C for 10 min. PCR reactions were performed in triplicate 20 μL mixture containing 4 μL of 5 × FastPfu Buffer, 2 μL of 2.5 mM dNTPs, 0.8 μL of each primer (5 μM), 0.4 μL of FastPfu Polymerase, and 10 ng of template DNA. The resulted PCR products were extracted from a 2% agarose gel and further purified using the AxyPrep DNA Gel Extraction Kit (Axygen Biosciences, Union City, CA, USA) and quantified using QuantiFluor™-ST (Promega, USA) according to the manufacturer’s protocol. Purified amplicons were pooled in equimolar and paired-end sequenced (2 × 300) on an Illumina MiSeq platform (Illumina, San Diego, USA) according to the standard protocols by Majorbio Bio-Pharm Technology Co. Ltd. (Shanghai, China). Raw fastq files were demultiplexed, quality-filtered by Trimmomatic, and merged by FLASH with the following criteria: (a) The reads were truncated at any site receiving an average quality score < 20 over a 50-bp sliding window. (ii) Primers were exactly matched allowing two nucleotide mismatching, and reads containing ambiguous bases were removed. (iii) Sequences whose overlap longer than 10 bp were merged according to their overlap sequence. Operational taxonomic units (OTUs) were clustered with 97% similarity [75] cutoff using UPARSE (version 7.1 http://drive5.com/uparse/) and chimeric sequences were identified and removed using UCHIME. The taxonomy of each 16S rRNA gene sequence was analyzed by RDP Classifier algorithm (http://rdp.cme.msu.edu/) against the Silva (SSU123) 16S rRNA database using a confidence threshold of 70%.

### Shotgun metagenomics of cecum microbiota

A total of 12 samples (CON1–6, OTA1–6) were selected for shotgun metagenomics sequencing. Using Nextra XT protocols (Illumina), individual libraries were sequenced on the Miseq platform. 3′ and 5′ ends were stripped using SeqPrep (https://github.com/jstjohn/SeqPrep). Low-quality reads (length < 50 bp or with a quality value < 20 or having N bases) were removed by Sickle (https://github.com/najoshi/sickle). Reads were aligned to the *Anas platyrhynchos (mallard)* (GenBank assembly accession: GCA_000355885.1 and RefSeq assembly accession: GCF_000355885.1) by BWA (http://bio-bwa.sourceforge.net) and any hit associated with the reads and their mated reads were removed. De bruijn-graph-based assembler SOAPdenovo (http://soap.genomics.org.cn, version 1.06) was employed to assemble short reads. K-mers, varying from 1/3~2/3 of reads length, were tested for each sample. Scaffolds with a length over 500 bp were retained for statistical tests; we evaluated the quality and quantity of scaffolds generated by each assembly and finally chose the best K-mer which yielded the minimum scaffold number and the maximum value of N50 and N90. Then, scaffolds with a length of over 500 bp were extracted and broken into contigs without gaps. Contigs were used for further gene prediction and annotation.

### Gene prediction, taxonomy, and functional annotation

Open reading frames (ORFs) from each metagenomic sample were predicted using MetaGene (http://metagene.cb.k.u-tokyo.ac.jp/). The predicted ORFs with length being or over 100 bp were retrieved and translated to amino acid sequences using the NCBI translation table (http://www.ncbi.nlm.nih.gov/Taxonomy/taxonomyhome.html/index.cgi?chapter=tgencodes#SG1). All sequences from gene sets with a 95% sequence identity (90% coverage) were clustered as the non-redundant gene catalog by the CD-HIT (http://www.bioinformatics.org/cd-hit/). Reads after quality control were mapped to the representative genes with 95% identity using SOAP aligner (http://soap.genomics.org.cn/), and gene abundance in each sample was evaluated. BLASTP (version 2.2.28+, http://blast.ncbi.nlm.nih.gov/Blast.cgi) was employed for taxonomic annotations by aligning non-redundant gene catalogs against NCBI NR database with e-value cutoff of 1e^−5^. The cluster of orthologous groups of proteins (COG) for the ORF annotation was performed using BLASTP against eggNOG database (v4.5) with an e-value cutoff of 1e^−5^. The KEGG pathway annotation was conducted using BLASTP search (version 2.2.28+) against the Kyoto Encyclopedia of Genes and Genomes database (http://www.genome.jp/keeg/) with an e-value cutoff of 1e^−5^.

### Transcriptional analysis

Total RNA was isolated from liquid nitrogen-frozen liver and cecum mucus using the Quick-RNA™ MiniPrep Plus (Zymo, USA) according to the manufacturer’s instructions. The synthesis of the first strand (cDNA) was performed using oligo (dT) 20 and Superscript II reverse transcriptase (Takara, Japan). The transcriptional analysis of the related genes were performed using the following primers: *TJP1:* 5′- TCA GCG AGA TGA ACG AGC C-3′, 5′- TCT GAA GGC TCT GAC CTC TGG-3′, *OCLN:* 5′- GCT GGG CTA CAA CTA CGG GT-3′, 5′- TAC GCC AAC ACG GTG CTG-3′, *TLR4*: 5′- TTA ACT GCC AAT TTG CTC C-3′, 5′- CCG GTT TCC ACC AAT ACT A-3′, *MYD88*: 5′- 5'-GAAGAGGAAGCAGCAGCAA-3', 5'-TGAACCGCAGGATACTTGG-3', *IKB-α*: 5′- CGT GTC TCC ATT TGG CAT CT-3′, 5′- GCC CTG GTA GGT CAC TTT GT-3′, *TNF-α*: 5′- ACA GCC TAT GCC AAC AAG-3′, 5′- TAC AGG AAG GGC AAC TCA-3′, *IL-6*: 5′- AAA GCA TCT GGC AAC GAC-3′, 5′- AAT AGC GAA CAG CCC TCA-3′. *β-Actin* (5′- TAC GCC AAC ACG GTG CTG-3′, 5′- GAT TCA TCA TAC TCC TGC TTG-3′) was used as an internal control to normalize target gene transcriptional levels.

### Statistical analysis

The data are expressed as mean ± SEM and analyzed using GraphPad Prism 6.0 (GraphPad Software). Significant differences between the two groups were evaluated by two-tailed unpaired Student’s *t* test or Mann-Whitney *U* test for samples that were not normally distributed. Significant differences among three or more groups were evaluated by one-way ANOVA with Bonferroni’s multiple comparisons test. The level of significance was set at *P* < 0.05; *, *P* < 0.05; **, *P* < 0.01; ***, *P* < 0.001; ****, *P* < 0.0001.

## Supplementary information

**Additional file 1: Figure S1.** OTA residue and effect of OTA on growth performance and organ index in ducks. **a:** OTA residue in different organs (*n* = 6, Mean with SEM). The different letters above the column show significant differences. **b-e:** Effect of OTA on growth performance of ducks from day 1 to day 21. **f:** Effect of OTA on relative organ weight of 21 d ducks (n=6, Mean with SEM). **g:** Effect of OTA on relative length of intestine on day 21 ducks (n=6, Mean with SEM). **h:** Effect of OTA on relative weight of intestine on day 21 ducks. Data in **a** were analyzed by one-way ANOVA with Bonferroni’s multiple comparisons test, while data in **b-h** were analyzed by unpaired t test, *P <0 .05.

**Additional file 2: Figure S2.** OTA alters cecum microbial diversity and composition. **a:** Alpha diversity of cecum microbiota in different groups (n=7). Data were analyzed by student’s t test, *** *P*<0.001. **b:** Relative abundance of bacteria at Phylum level. OTUs with an occurrence lower than 1% are not represented (n=7). **c:** Relative abundance of top 15 Genus in each group (n=7). **d:** Relative abundance of top 15 Species in each group (n = 7).

**Additional file 3: Figure S3.** OTA lowers intestinal abundance of tight junctions. **a:** Effect of OTA on relative mRNA expressions of TJP1 and Occludin in the cecum of 21d ducks (n=6, Mean with SEM). Data were analyzed by unpaired t test. **b:** Effect of OTA on the protein abundance of TJP1 and Occludin in the cecum (n=6, Mean with SEM). Data were analyzed by unpaired t test. (n=6). *P<0 .05, **P<0 .01, *** *P*<0.001.

**Additional file 4: Figure S4.** OTA alters liver microbial composition. **a:** Alpha diversity of liver microbiota in different groups (n=5). Data were analyzed by student’s t test, n.s., not significant. **b:** Relative abundance of bacteria at Phylum level. OTUs with an occurrence lower than 1% are not represented (n=5). **c:** Relative abundance of top 15 Genus in each group (n=5). **d:** Relative abundance of top 15 Species in each group (n=5).

**Additional file 5: Figure S5.** OTA residue, effect of OTA on growth performance and organ index in antibiotics-treated ducks. **a:** OTA residue in different organs (n=6, Mean with SEM). Data were analyzed by the one-way ANOVA followed by Dunnett multiple comparisons (n=6, Mean with SEM). The different letters above the column show significant differences. **b-e:** Effect of OTA on growth performance of antibiotics-treated ducks from day 1 to day 21. **f:** Effect of OTA on relative organ weight of antibiotics-treated ducks (n=6, Mean with SEM). **g:** Effect of OTA on relative length of intestine on antibiotics-treated ducks (n=6, Mean with SEM). **h:** Effect of OTA on relative weight of intestine on antibiotics-treated ducks. Data in **b-h** were analyzed by unpaired t test.

**Additional file 6: Figure S6.** OTA has little effect on cecum microbiota diversity and composition in antibiotics-treated ducks. **a:** Alpha diversity of liver microbiota in different groups (n=6, Mean with SEM). **b:** The OTU numbers of *Bacteroidetes* (left) and *Bacteroides* (right) in different groups (CON, n=10; Antibiotics, n=6; Anti+OTA, n=6, mean with SEM). **c:** Relative abundance of top 7 bacteria at Phylum level in antibiotics-treated ducks. **d:** Relative abundance of top 15 bacteria at species level in antibiotics treated ducks. Data in **a** were analyzed by unpaired t test; while data in **b** were analyzed by one-way ANOVA with Bonferroni’s multiple comparisons test. n.s., not significant; ****P<0.0001.

**Additional file 7: Figure S7.** OTA shows little effect on intestinal abundance of tight junctions in antibiotics-treated ducks. **a:** Effect of OTA on relative mRNA expressions of TJP1 and Occludin in the cecum of antibiotics-treated ducks (n=6, Mean with SEM). Data were analyzed by unpaired t test. **b:** Effect of OTA on the protein abundance of TJP1 and Occludin in the cecum (n=6, Mean with SEM). Data were analyzed by unpaired t test. (n=6). *P<0 .05.

**Additional file 8: Figure S8.** OTA residue, growth performance and organ index after intestinal microbiota transplantation. **a:** OTA residue in the cecum digesta and liver of FMT (OTA) ducks (n=6, Mean with SEM). **b-c:** Final weight and relative weight of liver in FMT (CON) and FMT (OTA) ducks (n=9, mean with SEM). **d:** Levels of pro-inflammatory cytokines in duck liver after administration of OTA at dosage of 0, 15, 30 or 60 μg/kg (n=6, mean with SEM). Data in **a**, **b** and **c** were analyzed by unpaired t test; while data in **d** were analyzed by one-way ANOVA with Bonferroni’s multiple comparisons test. *P<0 .05. FMT (CON), ducks received CON group fecal microbiota; FMT (OTA), ducks received OTA group fecal microbiota.

**Additional file 9: Figure S9.** The cecum microbial diversity and composition of transplant donors and recipients. **a:** Alpha diversity of intestinal microbiome in transplant donors and recipients. **b:** Relative abundance of bacteria at Phylum level. OTUs with an occurrence lower than 1% are not represented (n=7). **c**: Relative abundance of top 15 Genus in each group (n=7). **d:** Relative abundance of top 15 Species in each group (n=7). Data in **a** and **b** were analyzed by one-way ANOVA with Bonferroni’s multiple comparisons test *P<0.05. FMT (CON): ducks received CON group fecal microbiota. FMT (OTA): ducks received OTA group fecal microbiota.

**Additional file 10: Figure S10.** OTA-originated microbiota lowers intestinal abundance of tight junctions. **a:** Relative mRNA expression of TJP1 and Occludin in different groups (n=6). **b:** Relative protein abundance of TJP1 and Occludin (n=6). Data were analyzed by unpaired t test, n=6, mean with SEM. *P<0 .05, **** *P*<0.0001. FMT (CON): ducks received CON group fecal microbiota. FMT (OTA): ducks received OTA group fecal microbiota.

## Data Availability

All raw data of 16S rRNA amplicon sequencing and Shotgun metagenomics are available at Sequence Read Archive (Accession number SUB5539876).

## Abbreviations

ALP: Alkaline phosphatase
ALT: Alanine aminotransferase
AST: Aspartate aminotransferase
FMT: Fecal microbiota transplantation
LDH: Lactate dehydrogenase
LPS: Lipopolysaccharides
MAPKs: Mitogen-activated protein kinases
OTA: Ochratoxin A
PRRs: Pattern-recognizing receptors
TLR4: Toll-like receptor 4
