# Supplementary figures and images for: Ochratoxin A induces liver inflammation: involvement of intestinal microbiota
Source: Microbiome. 2019 Nov 28;7:151. doi: 10.1186/s40168-019-0761-z (PMC6883682; doi:10.1186/s40168-019-0761-z)

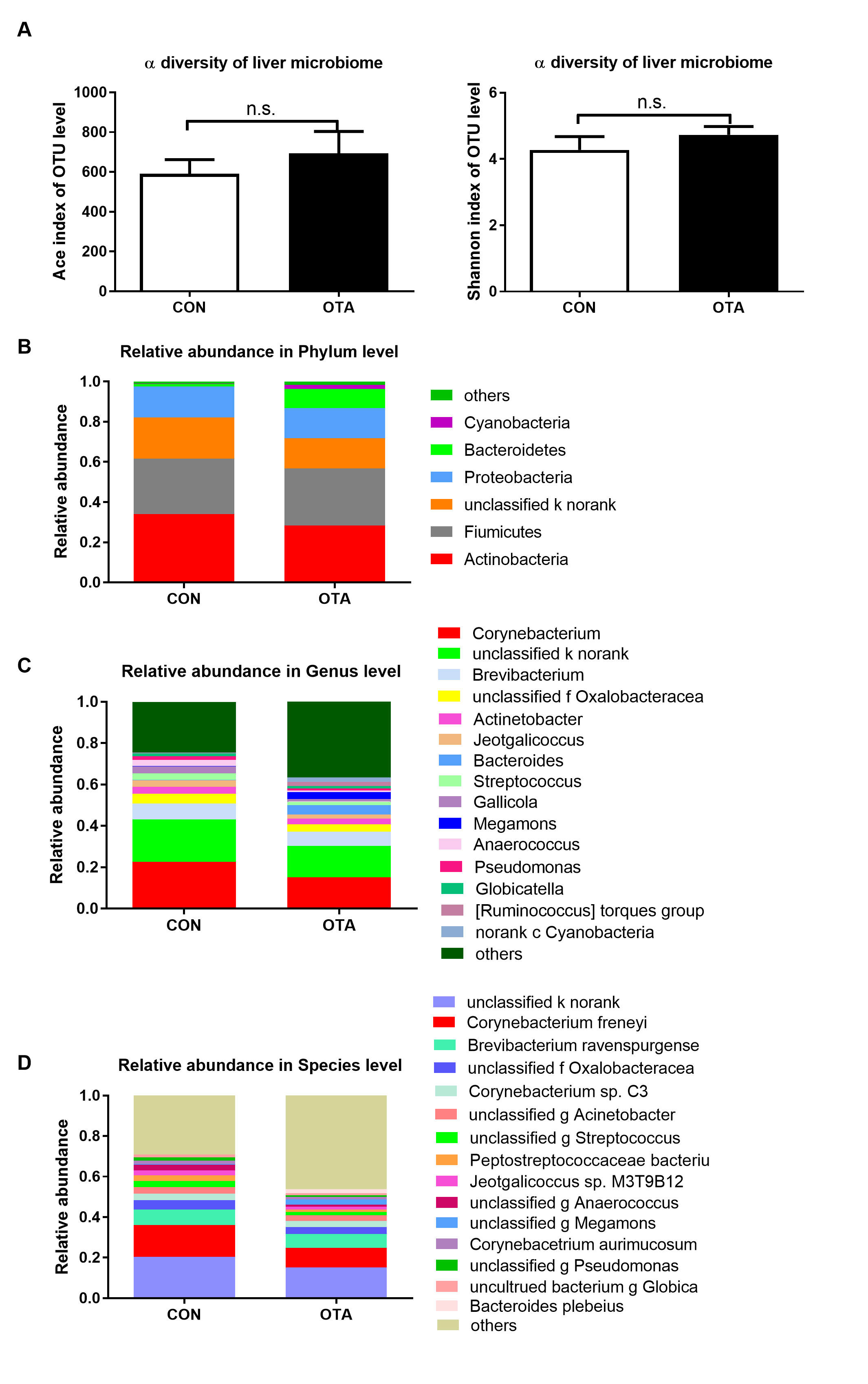

Supplement: Supplementary file 4 — Additional file 4: Figure S4. OTA alters liver microbial composition. a: Alpha diversity of liver microbiota in different groups (n=5). Data were analyzed by student’s t test, n.s., not significant. b: Relative abundance of bacteria at Phylum level. OTUs with an occurrence lower than 1% are not represented (n=5). c: Relative abundance of top 15 Genus in each group (n=5). d: Relative abundance of top 15 Species in each group (n=5). [file 40168_2019_761_MOESM4_ESM.tif]

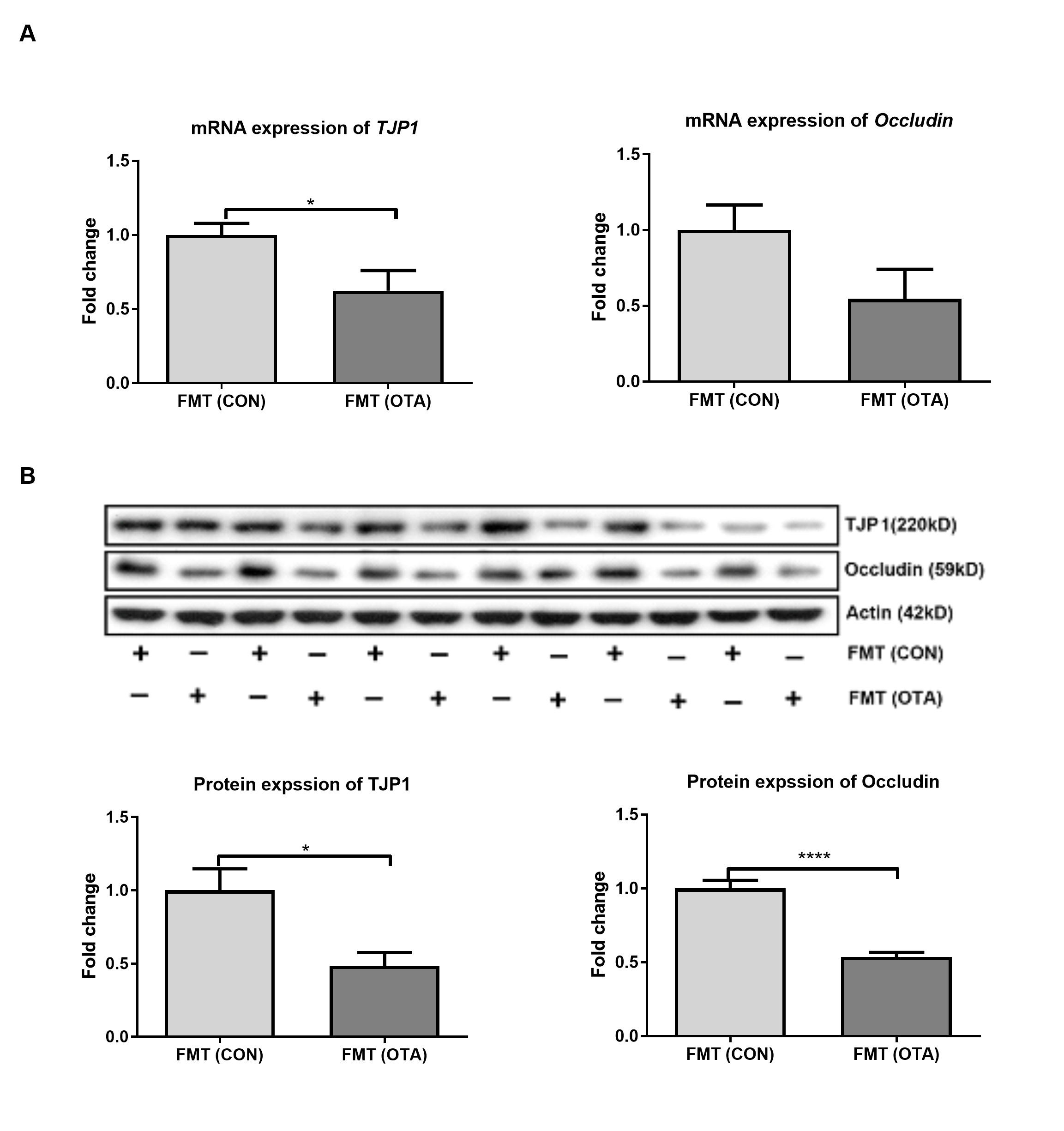

Supplement: Supplementary file 10 — Additional file 10: Figure S10. OTA-originated microbiota lowers intestinal abundance of tight junctions. a: Relative mRNA expression of TJP1 and Occludin in different groups (n=6). b: Relative protein abundance of TJP1 and Occludin (n=6). Data were analyzed by unpaired t test, n=6, mean with SEM. *P<0 .05, **** P<0.0001. FMT (CON): ducks received CON group fecal microbiota. FMT (OTA): ducks received OTA group fecal microbiota. [file 40168_2019_761_MOESM10_ESM.tif]
